# A 2-year multimodal prospective cohort study to predict sustained progression independent of relapse activity in multiple sclerosis

**DOI:** 10.3389/fneur.2026.1902455

**Published:** 2026-10-13

**Authors:** Luis Rafael Solís-Tarazona, Salma Reddam, Ana Merchán-Cabanillas, Silvia Navarro-Quevedo, Lars Lau Raket, Sara Gil-Perotin

**Affiliations:** 1 Research Group in Immunotherapy and Biomodels for Autoimmunity, Instituto de Investigación Sanitaria La Fe, Valencia, Spain; 2 Department of Neurology, Hospital Dr. Peset, Valencia, Spain; 3 Department of Neurology, Hospital Universitario Vega Baja, Alicante, Spain; 4 Clinical Memory Research Unit, Department of Clinical Sciences, Lund University, Lund, Sweden; 5 Division of Hematology and Oncology, Department of Medicine, Columbia Initiative in Cell Engineering and Therapy (CICET), Columbia University Irving Medical Center, New York, NY, United States

**Keywords:** biomarkers, CHI3L1, fatigue, MFIs, multimodal, multiple sclerosis, PIRA, smoldering MS

## Abstract

**Background:**

Disability accrual in multiple sclerosis (MS) is heterogeneous. We assessed whether a 2-year multimodal evaluation could identify patients who were developing progression independent of relapse activity (PIRA) or relapse-associated worsening (RAW).

**Methods:**

Among the 81 MS patients with a 2-year follow-up and outcomes determined through 5 years, a composite for PIRA was developed using Firth’s penalized logistic regression analysis; discrimination was calculated as the area under the curve (AUC) using repeated stratified nested cross-validation.

**Results:**

In total, 21 patients (25.9%) met the PIRA criteria, 9 (11.1%) met the RAW criteria, and 1 met both criteria. A multivariate Firth regression analysis selected two variables associated with PIRA after adjustment: Modified Fatigue Impact Scale (MFIS) total score (OR 2.04, 95% CI 1.17–3.83; *p* = 0.011) and cerebrospinal fluid (CSF) chitinase-3-like protein 1 (CHI3L1) (OR 2.00, 95% CI 1.11–5.26; *p* = 0.018). Notably, the Symbol Digit Modalities Test (ΔSDMT) showed an association in the univariate analysis (OR 0.47, 95% CI 0.23–0.82; *p* = 0.006) but was attenuated in the multivariate analysis [odds ratio (OR) 0.64; *p* = 0.141]. Oligoclonal immunoglobulin G band (OCB) positivity was universal in PIRA, but the findings are inconclusive due to sparse OCB-negative observations and incomplete OCB data. Worsening gait [ΔTimed 25-Foot Walk (ΔT25FW)] and new T2 lesions showed associations in the univariate analysis that were not retained in the multivariate analysis. None of the variables had a *p*-value of less than 0.10 for RAW. The median out-of-sample AUC was 0.74 (95% CI 0.68–0.78).

**Conclusion:**

A two-year multimodal assessment identified a biologically coherent PIRA composite driven by intrathecal neuroinflammation and fatigue. No reliable RAW predictor was found, and external validation is warranted.

## Introduction

Progressive disability is the main determinant of long-term outcomes in multiple sclerosis (MS) and a central endpoint in clinical practice and research. Conventional monitoring relies on relapses, inflammatory activity on magnetic resonance imaging (MRI), and changes in the Expanded Disability Status Scale (EDSS) (1) with standardized clinical course descriptors framing stratification and trial design (2). These measures do not fully capture the gradual, heterogeneous processes driving irreversible worsening.

Disability accrual may follow mechanistically distinct routes, either in close temporal association with relapses (relapse-associated worsening, RAW) or independently of acute inflammation (progression independent of relapse activity, PIRA) (3, 4). PIRA occurs even in relapsing–remitting MS from the earliest stages and accounts for the majority of disability accumulation (5). Both can coexist within the same patient, but they differ in their underlying biology, clinical correlates, and disease-modifying therapy (DMT) responsiveness. Distinguishing them prospectively has direct implications for monitoring and treatment.

Integrated strategies combining repeated disability measurements; functional performance tests, such as the Timed 25-Foot Walk (T25FW), the Nine-Hole Peg Test (9HPT), and the Symbol Digit Modalities Test (SDMT); and biological markers may improve prospective identification. Serum neurofilament light chain (sNfL) is a robust marker of neuroaxonal damage and prognostication (6), while glial markers, such as cerebrospinal fluid (CSF) chitinase-3-like protein 1 (CHI3L1) and serum GFAP, reflect astrocytic and microglial activation and are associated with progression-related disability from early stages (7, 8). Combined neuroaxonal and glial profiling supports phenotype characterization and prognostication, with complementary value at onset for predicting RAW and PIRA (9–11). Clinically applicable tools integrating these markers into a unified predictive model for sustained PIRA in early MS remain lacking.

In this prospective study, we used a structured 2-year follow-up incorporating clinical, functional, patient-reported, and fluid biomarker assessments, with extended observation up to 5 years for outcome confirmation, to develop and evaluate a composite predictive model for sustained PIRA.

## Methods

### Study design and setting

This prospective, longitudinal, observational study included consecutive patients according to a predefined clinical protocol at a tertiary MS referral center; data were collected during routine care and were recorded in a structured research database. The primary analytical window was 2 years, with extended observation up to 5 years for outcome confirmation. The protocol was approved by the Research Ethics Committee of Hospital Universitari i Politècnic La Fe, Valencia (2017/0476, PIIDPEMIT, 2023-1,077-1), which followed the principles of the Declaration of Helsinki; all participants provided written informed consent.

Of the 129 enrolled patients, 81 with a complete 2-year functional follow-up were included in the analytical cohort. Recruitment was not restricted to diagnostic onset to ensure an adequate sample size. Patients were eligible if they met the following criteria: age ≤45 years at baseline; confirmed relapsing MS per the 2017 McDonald criteria (12); baseline clinical, functional, and biomarker assessments; a ≥ 2-year follow-up with repeated disability evaluations; and longitudinal records permitting outcome confirmation. Patients with secondary or primary progressive MS at baseline were excluded from the study.

### Clinical definitions

A relapse was defined as new or recurrent, MS-attributable neurological symptoms lasting ≥24 h without fever or infection, separated from prior relapses by ≥30 days. Radiological activity was defined as new or enlarging T2 lesions and/or gadolinium-enhancing T1 lesions. RAW required an EDSS increase within 90 days after or 30 days before a relapse, meeting thresholds (≥1.5 if baseline EDSS = 0; ≥1.0 if 1.0 to <5.5; ≥0.5 if ≥5.5), and sustained at confirmation ≥3 months later (3). Sustained PIRA required meeting the same thresholds in the absence of relapses (none within 90 days before or 30 days after) and persistence at all subsequent assessments without reversion (3). One patient fulfilled both criteria on separate occasions and was included in both analyses.

### Variables and biomarkers

Demographic and clinical variables included age, sex, age at onset, disease duration, and baseline DMT (moderate or high efficacy). The modified Rio (mRio) score (13) was calculated at baseline. Functional performance was assessed at baseline and follow-up using the SDMT in alternate forms across visits to mitigate practice effects, the 9HPT (dominant and non-dominant), the T25FW, and the EDSS. Longitudinal change was summarized as Δ (last − baseline). The cohort was previously compared cross-sectionally to a retrospective progressive MS cohort for patient-reported outcomes (14). CSF samples were obtained during diagnostic lumbar puncture, generally contemporaneous with the diagnosis of MS during a relapse and before DMT initiation (Supplementary Figure S1); the samples were stored following standard procedures at −80 °C in the Biobank La Fe. CSF NfL and CHI3L1 were measured using the neurofilament light enzyme-linked immunosorbent assay (NF-light) ELISA (UmanDiagnostics) and Quantikine ELISA (R&D Systems). OCBs were identified using isoelectric focusing with IgG-specific immunodetection in paired CSF and serum (≥2 CSF-restricted bands). Serum NfL was measured at baseline and follow-up (NF-light Advantage, Simoa SR-X, Quanterix); derived metrics included baseline, mean, maximum, and ΔsNfL. MRI of the brain and spinal cord (standardized T1, T2, Fluid-attenuated inversion recovery (FLAIR), pre-gadolinium, and post-gadolinium) was performed at baseline and yearly; follow-up activity was summarized as cumulative new T2 and gadolinium-enhancing lesion counts and binary indicators. Patient-reported outcomes included Modified fatigue impact scale (MFIS) total and subscores and Beck Depression Inventory-II (BDI-II); they were administered at 6 months (V2) and were therefore considered longitudinal markers of progression rather than baseline predictors of progression.

### Composite model development and statistical analysis

Predictive models for RAW and PIRA used two-step variable selection. Each candidate was screened using a Firth-penalized logistic regression analysis after adjusting for age and disease duration (threshold *p* < 0.10); selected predictors entered into a multivariable Firth model with age and disease duration as mandatory covariates. The procedure was repeated within each training fold of a nested cross-validation. Firth’s penalization was used to reduce small-sample bias and yield finite estimates under quasi-complete separation. All patients with PIRA events and available OCB data were OCB-positive. The candidate predictors comprised baseline EDSS, Δ functional measures (ΔSDMT, Δ9HPT dominant and non-dominant, and ΔT25FW), baseline CSF NfL and CHI3L1, serum NfL (baseline, mean, maximum, and ΔsNfL), OCB positivity, MFIS total and BDI at V2, myelitis, and total and any new T2 lesions during follow-up. Continuous predictors were standardized to one SD; missing data were median-imputed (Supplementary Table S1). Continuous variables are reported as median [IQR], and categorical variables are reported as *n* (%). Comparisons used Wilcoxon rank-sum and Fisher’s exact tests (two-sided; *p* < 0.05). Discrimination was estimated as the AUC using a repeated stratified nested cross-validation (5-fold outer; full variable selection in the inner loop; 20 repeats), reported as median [2.5th–97.5th percentile]. Final ORs with 95% CIs were obtained from the multivariable Firth model using the complete cohort. Analyses were performed using R 4.4.1 with the logistf [logistf] and pROC [pROC] packages.

## Results

### Study population

Of the 129 consecutively enrolled patients, 81 with a complete two-year functional follow-up were included in the primary analysis cohort. In total, 21 patients (25.9%) fulfilled the sustained PIRA criteria, and 9 (11.1%) fulfilled the RAW criteria; one patient met both on separate occasions and is described separately. The remaining 52 patients met neither outcome criterion. The annualized relapse rate (ARR) was relatively low among the complete 2-year population (0.141). Only six patients (7.4%) were newly diagnosed with secondary progressive MS (SPMS) during the study.

### Baseline characteristics and longitudinal changes by disease status

Baseline characteristics by outcome group are shown in Table **1**. A table with the baseline characteristics of the population included (*N* = 81) and excluded (*N* = 48) in the analysis can be found in Supplementary Table S1. OCB data were available for 38 of the 52 stable patients, 17 of the 20 patients in the PIRA group, and 4 of the 8 patients in the RAW group. Patients who experienced PIRA had significantly higher baseline CSF CHI3L1than stable patients [median 197 (124, 332) vs. 119 (84, 159) pg/mL; *p* = 0.010], universal OCB positivity (17 of 17 vs. 29 of 38; *p* = 0.031), and substantially higher MFIS total at V2 [30 (26, 71) vs. 9 (5, 23); *p* = 0.003]. No baseline variable showed significant differences between the RAW and stable patients. When comparing the PIRA and RAW groups, DMT type differed (high-efficacy 90% vs. 38%; *p* = 0.009) as did OCB positivity (17 of 17 vs. 2 of 4; *p* = 0.004), although the small size of the RAW group limits the interpretation of this finding. Age, sex, disease duration, baseline EDSS, myelitis, mRio score, aHSCT, and BDI-II did not differ between the PIRA and stable groups. Baseline distributions of variables by PIRA status are shown in Figures 1A,D.

**Table 1:** Baseline characteristics stratified by sustained outcome group.

Baseline variable	Stable (*N* = 52)	PIRA (*N* = 20)	RAW (*N* = 8)	*p* (PIRA vs. stable)	*p* (RAW vs. stable)	*p* (PIRA vs. RAW)
Sex				0.76	0.43	1.00
Female	39 (75%)	14 (70%)	5 (63%)			
Male	13 (25%)	6 (30%)	3 (38%)			
Age at baseline (years)	31.0 [27.0, 37.0]	32.0 [29.0, 34.5]	34.5 [27.5, 39.0]	0.79	0.29	0.50
Disease duration (months)	54 [33, 87]	80 [45, 103]	84 [27, 113]	0.16	0.54	0.90
EDSS category				0.75	0.32	0.27
<3	42 (81%)	15 (75%)	8 (100%)			
≥3	10 (19%)	5 (25%)	0 (0%)			
Myelitis at onset				0.79	0.71	1.00
No	19 (37%)	6 (30%)	2 (25%)			
Yes	33 (63%)	14 (70%)	6 (75%)			
CSF CHI3L1 (pg/mL)	119 [84, 159]	197 [124, 332]	135 [125, 152]	0.010	0.52	0.15
OCBs	29 (76%)	17 (100%)	2 (50%)	0.031	0.27	0.004
mRio score				0.88	0.95	1.00
0	40 (77%)	14 (74%)	6 (75%)			
1	5 (9.6%)	3 (16%)	1 (13%)			
2	6 (12%)	2 (11%)	1 (13%)			
3	1 (1.9%)	0 (0%)	0 (0%)			
DMT type				0.16	0.22	0.009
No DMT	1 (1.9%)	0 (0%)	0 (0%)			
High efficacy	36 (69%)	18 (90%)	3 (38%)			
Low/moderate	15 (29%)	2 (10%)	5 (63%)			
Alemtuzumab (ever)	12 (23%)	5 (25%)	0 (0%)	0.87	0.14	0.14
aHSCT	2 (3.8%)	2 (10%)	1 (13%)	0.32	0.31	0.89
MFIS total score (V2)	9 [5, 23]	30 [26, 71]	16 [6, 38]	0.003	0.36	0.10
BDI-II score (V2)	7 [3, 20]	17 [7, 26]	7 [1, 23]	0.27	0.93	0.52

Values are median [IQR] or n (%). Group comparisons used the Wilcoxon rank-sum test for continuous variables and Fisher’s exact test for categorical variables. Patients who fulfilled the criteria for both PIRA and RAW on separate occasions during follow-up were excluded from this three-group comparison and are described separately. OCB data were available for 38 of 52 stable patients, 17 of 20 PIRA patients, and 4 of 8 RAW patients; missing values were imputed for modeling purposes, but not for descriptive comparisons. aHSCT, autologous hematopoietic stem cell transplantation; CHI3L1, chitinase-3-like protein 1; CSF, cerebrospinal fluid; DMT, disease-modifying therapy; EDSS, Expanded Disability Status Scale; IQR, interquartile range; MFIS, Modified Fatigue Impact Scale; mRio, modified Rio score; NfL, neurofilament light chain; OCBs, oligoclonal IgG bands; PIRA, progression independent of relapse activity; RAW, relapse-associated worsening; V2, visit 2 (6-month follow-up).

**Figure 1:**
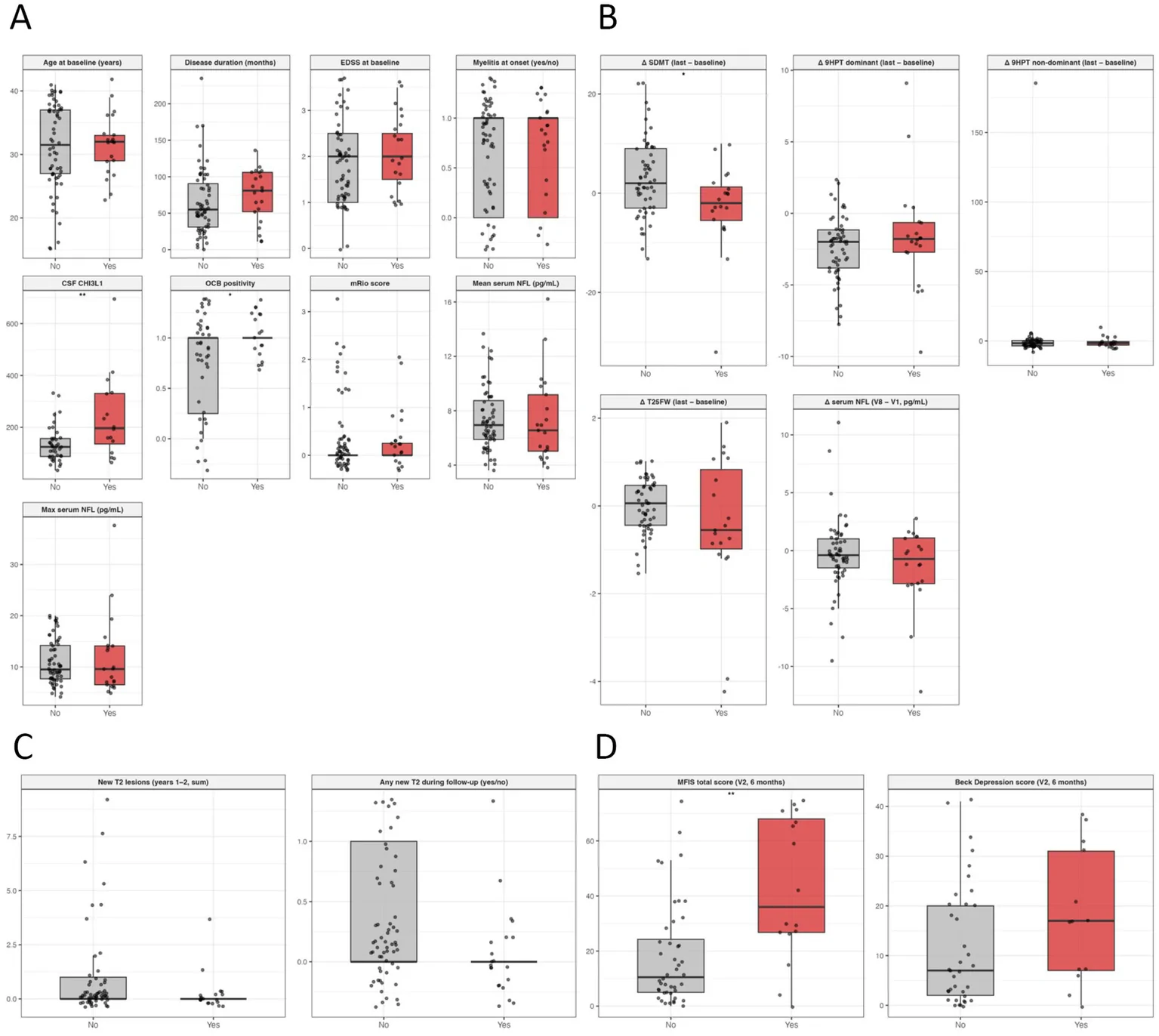
Distributions of variables by PIRA status (Wilcoxon rank-sum tests). Panel **(A)**: baseline clinical and biomarker variables, including age at baseline, disease duration, EDSS, OCB positivity, CSF CHI3L1, mean serum NfL, and myelitis at onset; Panel **(B)**: longitudinal changes (Δ; last visit minus baseline) in EDSS, SDMT, 9HPT (dominant and non-dominant), T25FW, and serum NfL within the two-year follow-up window; Panel **(C)**: MRI activity during follow-up (years 1 and 2), expressed as the cumulative number of new T2 lesions and the presence of any new T2 lesion (binary); Panel **(D)**: patient-reported outcomes at 6 months (V2), including MFIS total score with physical, cognitive, and psychosocial subscores, and BDI-II score. Asterisks indicate Wilcoxon *p*-values: ^***^p<0.001; ^**^p<0.01; ^*^p<0.05. Non-significant comparisons are not labelled. BDI-II, Beck Depression Inventory; CSF, cerebrospinal fluid; CHI3L1, chitinase-3-like protein 1; EDSS, Expanded Disability Status Scale; MFIS, Modified Fatigue Impact Scale; NfL, neurofilament light chain; OCBs, oligoclonal IgG bands; PIRA, progression independent of relapse activity; SDMT, Symbol Digit Modalities Test; T25FW, Timed 25-Foot Walk; 9HPT, Nine-Hole Peg Test.

Longitudinal changes are shown in Table 2. By definition, stable patients did not show a sustained EDSS increase, apart from transient, non-sustained variations, in contrast to the PIRA (*p* < 0.001) and RAW groups (*p* = 0.003). A+ 0.5 or +1 EDSS increase was observed in 12 of 20 (60%) patients with PIRA and in 5 of 8 (63%) of those with RAW events. Cognitive function worsened more in the PIRA group [ΔSDMT median −1 (−7, 2) vs. +2 (−1, 6) in stable patients; *p* = 0.027]. In the RAW group, the ΔSDMT was numerically higher than in stable patients [+9 (−4, 14) vs. +2 (−1, 6); *p* = 0.30] and than in those in the PIRA group (*p* = 0.07), although neither comparison reached statistical significance. Other functional measures (Δ9HPT dominant/non-dominant and ΔT25FW) and ΔsNfL did not differ across groups. The mean sNfL across follow-up was higher in the RAW group than in stable patients [9.14 (7.05, 10.48) vs. 6.64 (5.73, 8.05) pg/mL; *p* = 0.07], again without reaching statistical significance. The distributions of longitudinal changes by PIRA status are shown in Figures 1B,C.

**Table 2:** Longitudinal changes stratified by outcome group.

Δ (last—baseline)	Stable (*N* = 52)	PIRA (*N* = 20)	RAW (*N* = 8)	*p* (PIRA vs. stable)	*p* (RAW vs. stable)	*p* (PIRA vs. RAW)
ΔEDSS				<0.001	0.003	0.96
−1 or less	7 (14%)	2 (10%)	1 (13%)			
0	30 (59%)	3 (15%)	1 (13%)			
+0.5	3 (5.9%)	5 (25%)	3 (38%)			
+1	2 (3.9%)	7 (35%)	2 (25%)			
Other	9 (18%)	3 (15%)	1 (13%)			
ΔSDMT (points)	2 [−1, 6]	−1 [−7, 2]	9 [−4, 14]	0.027	0.30	0.07
ΔDominant 9HPT (s)	−1.97 [−3.42, −1.15]	−1.85 [−2.75, −0.66]	−3.96 [−4.46, −0.88]	0.64	0.40	0.42
ΔNon-dominant 9HPT (s)	−1.99 [−3.55, 0.12]	−1.92 [−3.06, −0.38]	−0.35 [−2.89, 1.51]	1.00	0.29	0.53
ΔT25FW (s)	0.09 [−0.45, 0.57]	−0.59 [−1.10, 0.59]	−0.20 [−0.31, 0.40]	0.10	0.52	0.33
ΔSerum NfL (pg/mL)	−0.50 [−1.49, 0.80]	−0.24 [−2.91, 1.20]	0.18 [−2.17, 2.24]	0.59	0.44	0.36
Mean serum NfL (pg/mL)	6.64 [5.73, 8.05]	6.52 [4.89, 9.70]	9.14 [7.05, 10.48]	0.76	0.07	0.24

Values are median [IQR] or *n* (%). Group comparisons used the Wilcoxon rank-sum test for continuous variables and Fisher’s exact test for categorical variables. Δ denotes change (last—baseline) within the two-year structured follow-up window. Patients who fulfilled the criteria for both PIRA and RAW on separate occasions during follow-up were excluded from this three-group comparison. 9HPT, Nine-Hole Peg Test; EDSS, Expanded Disability Status Scale; IQR, interquartile range; NfL, neurofilament light chain; PIRA, progression independent of relapse activity; RAW, relapse-associated worsening; SDMT, Symbol Digit Modalities Test; T25FW, Timed 25-Foot Walk.

The amount of missing data was significant for some variables, most importantly the CSF biomarkers at baseline [CSF NFL: 29 of 52 missing (35.8%); CSF CHI3L1: 25 of 56 missing (30.9%); IgG OCB: 21 of 60 missing (25.9%)] and PROs [MFIS total score: 21 of 60 missing (25.9%); BDI-II: 31 of 50 missing (38.3%)]. Information about missing data in the included cohort can be found in Supplementary Table S2. Additional details on missing data for the population not included in the analysis can be found in Supplementary Table S1.

### Predictors of sustained PIRA and RAW

Univariate Firth penalized logistic regression models (each predictor adjusted for age and disease duration) identified six variables associated with sustained PIRA at the prespecified screening threshold (*p* < 0.10): MFIS total score at V2 [OR 2.33 (95% CI 1.42, 4.08); *p* < 0.001], baseline CSF CHI3L1 [OR 2.29 (95% CI 1.29, 4.67); *p* = 0.002], ΔSDMT [OR 0.47 (95% CI 0.23, 0.82); *p* = 0.006], OCB positivity [OR 10.0 (95% CI 1.21, upper bound not estimable due to complete separation); *p* = 0.029], ΔT25FW [OR 0.66 (95% CI 0.38, 1.04); *p* = 0.071], and any new T2 lesion during follow-up [OR 0.33 (95% CI 0.06, 1.20); *p* = 0.096]. Other variables (baseline EDSS, age, sex, mRio score, myelitis at onset, baseline sNfL, mean sNfL, maximum sNfL, ΔsNfL, Δdominant 9HPT, Δnon-dominant 9HPT, BDI score, and total new T2 lesion count) did not reach the screening threshold (Figure 2A).

**Figure 2:**
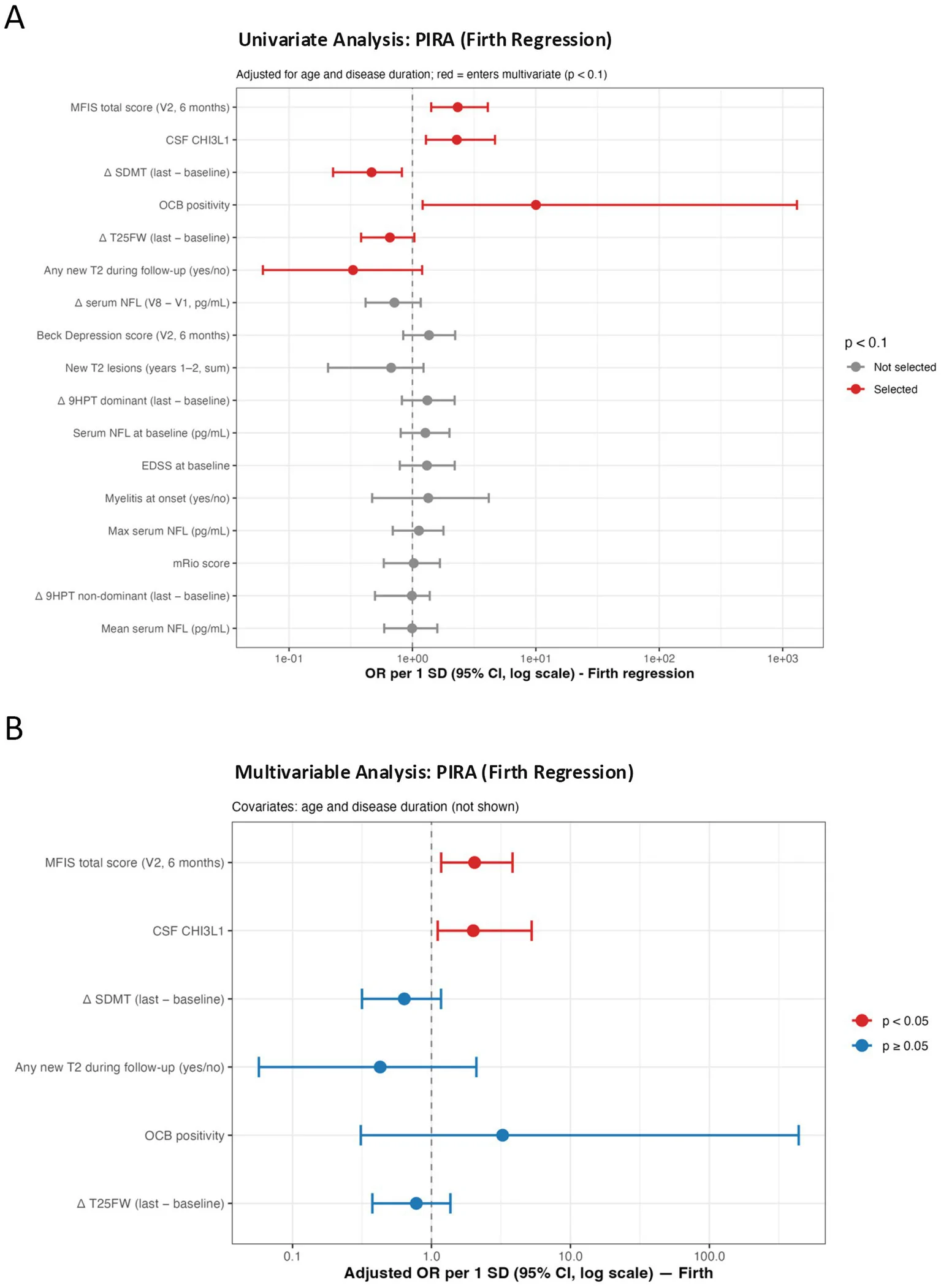
Forest plots of Firth penalized logistic regression models for sustained PIRA. Panel **(A)**: univariate analysis adjusted for age and disease duration. Variables shown in red passed the prespecified screening threshold (*p* < 0.10) and were entered into the multivariable model. Effect estimates are odds ratios (OR) per 1 standard deviation (SD) increase for continuous predictors or per category for binary predictors, with 95% confidence intervals on a log scale. The vertical dashed line indicates OR = 1 (no association); Panel **(B)**: multivariable model. The six variables that passed univariate screening were entered simultaneously into a multivariable Firth model, with age and disease duration as mandatory covariates. Variables in red reached statistical significance (*p* < 0.05); variables in blue did not. CSF, cerebrospinal fluid; CHI3L1, chitinase-3-like protein 1; EDSS, Expanded Disability Status Scale; MFIS, Modified Fatigue Impact Scale; NfL, neurofilament light chain; OCBs, oligoclonal IgG bands; SDMT, Symbol Digit Modalities Test; T25FW, Timed 25-Foot Walk; 9HPT, Nine-Hole Peg Test.

In the multivariable Firth model fitted on the complete analytical cohort, two variables retained statistical significance after adjustment: MFIS total score at V2 [OR 2.04 (95% CI 1.17, 3.83) per 1 SD increase; *p* = 0.011] and baseline CSF CHI3L1 [OR 2.00 (95% CI 1.11, 5.26) per 1 SD increase; *p* = 0.018]. The univariate associations between ΔSDMT [OR 0.64 (95% CI 0.32, 1.17); *p* = 0.141] and OCB positivity [OR 3.25 (95% CI 0.31, NE); *p* = 0.375] were attenuated after adjustment, reflecting partial overlap with the selected predictors (Figure 2B). Age, disease duration, ΔT25FW, and any new T2 lesion during follow-up did not reach significance in the adjusted model. Full results are shown in Table 3. The model assigned substantially higher predicted probabilities to patients who developed PIRA than to those who did not (Figure 3A). Discriminative performance of the multivariable model was evaluated through a repeated, stratified, nested cross-validation (*n* = 81; 21 events; 20 repeats) and yielded a median AUC of 0.74 (2.5th to 97.5th percentile interval: 0.68–0.78), reflecting moderate-to-good discrimination, with variable selection performed entirely within each training fold (Figure 3B). An internal cross-validation sensitivity analysis was performed and is presented in Supplementary Table S3.

**Table 3:** Results of the Firth logistic regression for sustained PIRA.

Step	Variable	OR (95% CI)	*p*-value
Univariate (selected: *p* < 0.10)	MFIS total score (V2)	2.33 (1.42, 4.08)	<0.001
	CSF CHI3L1	2.29 (1.29, 4.67)	0.002
	ΔSDMT	0.47 (0.23, 0.82)	0.006
	OCB positivity	10 (1.21, NE^a^)	0.029
	ΔT25FW	0.66 (0.38, 1.04)	0.071
	Any new T2 lesions during follow-up	0.33 (0.06, 1.20)	0.096
Univariate (not selected: *p* ≥ 0.10)	EDSS at baseline	1.31 (0.79, 2.20)	0.295
	Baseline serum NfL	1.27 (0.80, 1.99)	0.293
	Mean serum NfL	0.99 (0.59, 1.59)	0.976
	Maximum serum NfL	1.13 (0.69, 1.78)	0.601
	ΔsNfL	0.71 (0.42, 1.17)	0.182
	ΔDominant 9HPT	1.32 (0.82, 2.20)	0.248
	ΔNon-dominant 9HPT	0.99 (0.50, 1.38)	0.955
	Total new T2 lesions (years 1 and 2)	0.67 (0.21, 1.23)	0.239
	BDI-II score (V2)	1.36 (0.84, 2.22)	0.205
	Myelitis at onset	1.15 (0.70, 1.97)	0.587
	mRio score	1.02 (0.59, 1.67)	0.928
Multivariable	MFIS total score (V2)	2.04 (1.17, 3.83)	0.011
	CSF CHI3L1	2.00 (1.11, 5.26)	0.018
	Any new T2 during follow-up	0.43 (0.06, 2.10)	0.314
	ΔT25FW	0.78 (0.38, 1.37)	0.398
	OCB positivity	3.25 (0.31, NE^1^)	0.375
	Age at baseline (years)	0.92 (0.83, 1.02)	0.108
	Disease duration (months)	1.00 (0.98, 1.02)	0.841

a NE, not estimable due to complete separation, as all PIRA patients with available data were OCB-positive at baseline.

**Figure 3:**
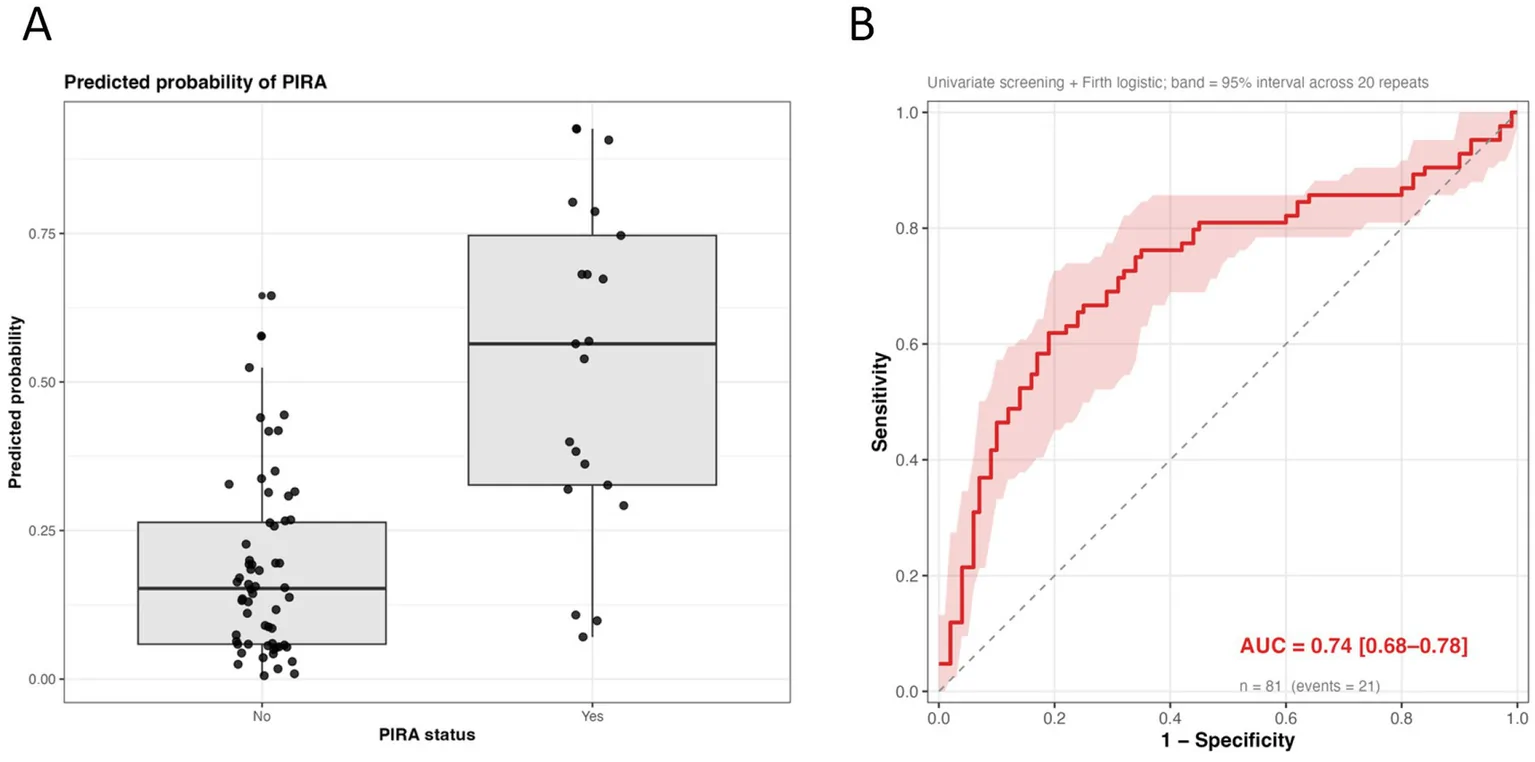
Predicted probabilities and out-of-sample discrimination of the PIRA composite model. Panel **(A)**: distribution of predicted probabilities of sustained PIRA stratified by observed outcome. Predicted probabilities were derived from the multivariable Firth penalized logistic regression model fitted on the complete analytical cohort, including all six predictors that passed univariate screening (MFIS total score at V2, CSF CHI3L1, ΔSDMT, OCB positivity, ΔT25FW, and any new T2 lesion during follow-up) with age and disease duration as mandatory covariates. Each point represents one patient. Boxes show median and IQR. The model assigns substantially higher predicted probabilities to patients who developed PIRA (median 0.43) than to those who did not (median 0.10). These are in-sample predictions; Panel **(B)**: out-of-sample receiver operating characteristic curve (ROC) obtained by repeated stratified nested cross-validation (*n* = 81; 21 events; 20 repeats). The bold line shows the median ROC across repeats; the shaded band represents the 2.5th to 97.5th percentile interval, reflecting variability from random fold assignment. The dashed diagonal indicates chance discrimination. AUC, area under the curve; CHI3L1, chitinase-3-like protein 1; CSF, cerebrospinal fluid; IQR, interquartile range; MFIS, Modified Fatigue Impact Scale; OCB, oligoclonal IgG band; PIRA, progression independent of relapse activity; ROC, receiver operating characteristic; SDMT, Symbol Digit Modalities Test; T25FW, Timed 25-Foot Walk; V2, visit 2 (6-month follow-up).

For RAW events, no baseline or longitudinal variable reached the prespecified screening threshold in univariate Firth models; consequently, no multivariable model was constructed for this outcome.

## Discussion

In this prospective 2-year cohort study of 81 patients with relapsing MS and outcomes ascertained up to 5 years, sustained PIRA was independently predicted by an exploratory parsimonious composite of MFIS fatigue score and baseline CSF CHI3L1, with moderate-to-good discrimination in an internally cross-validated model (AUC 0.74, 95% CI 0.68–0.78). Despite 90% of the PIRA group receiving high-efficacy DMT, progression still occurred, illustrating residual smoldering activity that may not be fully addressed by current anti-inflammatory therapy. Approximately one-quarter of the population included in the analysis experienced 3-month-confirmed PIRA, which is comparable to other studies with similar follow-up and PIRA-time-window definitions (3, 15). No candidate variable reached the screening threshold for RAW events, which is consistent with stochastic relapse timing and the small RAW event count.

The MFIS total score at a single 6-month assessment, rather than its rate of change, was the strongest univariate marker of sustained PIRA in our cohort (OR 2.33 per SD; *p* < 0.001), and this remained significant after adjustment. The MFIS captures fatigue across physical, cognitive, and psychosocial domains; higher scores are associated with greater lesion load, gray matter atrophy, and reduced thalamic volume (16). In parallel, fatigue in progressive MS likely reflects axonal loss, impaired connectivity, and microglial activation (17). An earlier cross-sectional analysis by our group (14) documented higher MFIS across the relapsing-to-progressive spectrum and a correlation between the cognitive MFIS subscore and CSF CHI3L1. Patients already in an early progressive phase at enrollment may have reported higher fatigue, so MFIS may also reflect concurrent smoldering activity. Our results are complemented by recent work from Glanz et al. (18), who showed that fatigue and cognitive measures continue to worsen even in patients without EDSS-defined PIRA, supporting the idea that evaluating additional clinical dimensions, such as fatigue, may have prognostic value for disability progression. This finding should, nevertheless, not be interpreted as a prediction from study entry; instead, the MFIS may provide updated information on symptomatic burden and may capture early functional changes not fully represented by the EDSS. The prognostic value of MFIS should therefore be confirmed in larger cohorts in which the prediction window begins strictly after PRO ascertainment.

Cognitive decline (ΔSDMT) showed a strong univariate association with PIRA but was attenuated after adjustment for MFIS, reflecting partial overlap between fatigue and cognitive trajectories. Greater cognitive slowing predicting PIRA is consistent with the SDMT sensitivity to diffuse gray matter pathology, thalamic atrophy, and progressive cortical demyelination, which Boccia et al. (19) further linked to paramagnetic rim lesion burden in relapse-free MS.

CSF CHI3L1 (YKL-40) is a marker of glial activation and chronic intrathecal neuroinflammation, is secreted by activated astrocytes and microglia and is elevated in progressive MS (7, 20). Its effect estimate was stable across univariate and multivariable models, suggesting an inflammatory signal partially independent of functional and patient-reported measures. CHI3L1+ astrocytes have been demonstrated at the chronic active edges of smoldering lesions (7, 20, 21), with chronic active lesions, cortical demyelination, and meningeal inflammation increasingly recognized as pathological substrates driving PIRA (22). CSF CHI3L1 may partly reflect the same astrocytic activation that serum GFAP indexes systemically (9). Prior literature has been mixed, with earlier studies suggesting only weak predictive potential for conversion to secondary progressive MS (23), whereas more recent prospective and meta-analytic work supports an overall predictive role for CHI3L1 in progressive disease (24, 25). A practical advantage of CSF CHI3L1 and other CSF biomarkers is that they can be reliably quantified by standard ELISA, without requiring an ultrasensitive Simoa platform, making this approach pragmatically applicable across MS clinics for early PIRA risk stratification.

OCB positivity is common in MS and is part of the supportive McDonald diagnostic criteria, with population-level prevalence typically >85% (26). Moreover, previous studies have reported inconsistent associations between OCB status and subsequent disability progression (27, 28). The universal OCB positivity in our PIRA group partly reflects the diagnostic background rather than a finding specific to progression. OCB-negative MS has consistently been associated with a milder course and lower SPMS conversion risk (26). Additionally, meningeal lymphoid aggregates of clonally expanded CNS B and plasma cells are associated with subpial demyelination and accelerated disability (29), and Rosenstein et al. (30) showed that a kappa-free light chain index independently predicts PIRA in early relapsing MS. The OCB finding, however, is exploratory, and this observation should not be interpreted as evidence that OCB positivity independently predicts PIRA. Its attenuation after multivariable adjustment may reflect sparse-data instability, missingness, or shared information with other covariates.

sNfL did not reach the screening threshold as a predictive variable for sustained PIRA, consistent with sNfL primarily reflecting acute neuroaxonal damage rather than chronic smoldering activity. Inter-individual variability with age, body mass index (BMI), and renal function further limits the utility of single time-point sNfL (31), and our finding aligns with cross-sectional data showing similar values across DMT regimens in clinically stable patients (32). In cohorts heavily exposed to anti-CD20 DMT such as ours, inflammatory activity is largely suppressed; elevations are more likely to flag breakthrough relapses, JC virus reactivation (33), or gradual rises from aging and smoldering injury. The composite proposed here can be applied in standard MS clinics, whereas ultrasensitive sNfL quantification remains confined to specialized centers. The absence of a reliable RAW predictor reflects the stochastic nature of relapse timing, the dampening of relapse activity by high-efficacy DMT, and limited power with only nine events. PIRA events account for approximately twice as many disability-accrual events per 100 patient-years as RAW events, with RAW events progressively suppressed by high-efficacy DMT, whereas PIRA rates remain stable (34).

The high prevalence of high-efficacy DMT in the PIRA group (90% vs. 65% in non-PIRA patients) illustrates the limitations of anti-inflammatory therapy in preventing smoldering neurodegeneration, highlighting the treatment-progression gap (35, 36). This pattern likely reflects a selection effect, with higher-risk patients being escalated to high-efficacy DMT yet still progressing. Cree et al. (4) showed that silent progression occurs even in patients meeting NEDA criteria, accounting for the majority of confirmed disability accumulation under high-efficacy treatment. Tur et al. (5) showed that early PIRA is established from the first demyelinating event and contributes to long-term disability regardless of subsequent escalation. The ability to identify patients at risk of sustained PIRA early carries growing therapeutic relevance, as emerging strategies targeting CNS-compartmentalized inflammation and chronic neurodegeneration enter clinical evaluation. Brain-penetrant BTK inhibitors and B-cell-directed cellular therapies, currently under investigation in progressive disease, are designed to reach intrathecal compartments that conventional anti-inflammatory DMTs do not effectively address. Composite predictors of sustained PIRA could help enrich future trials of these agents with patients most likely to benefit.

Our study has limitations. PIRA was defined using a 3-month window; studies using this time window have been previously reported in the literature (3, 15), but recent reviews have suggested a potential for the overestimation of PIRA using cutoffs below 6 or 12 months (37). The cohort size was small, there was clinical heterogeneity (age, disease duration, and DMT-type use), and the number of outcome events was modest; although Firth regression mitigates small-sample bias, confidence intervals remain wide, and predictor selection is exploratory. As a single-center tertiary referral study, the cohort is enriched for complex disease and high-efficacy DMT, limiting generalizability. Of the 129 enrolled patients, 48 were not included in the analytical cohort because no functional assessment was available at the 24-month visit, following an interruption in the systematic administration of the performance battery that affected a defined period of study conduct. These patients remained under clinical follow-up, with the same median number of EDSS assessments as those included, and the exclusion was determined by the calendar of data collection rather than by clinical characteristics. They were nonetheless at an earlier stage of disease than the analytical cohort, with shorter disease duration, lower baseline EDSS, and lower baseline CSF CHI3L1, so our findings should be interpreted cautiously when extrapolated to patients at the earliest stages of relapsing MS. We also acknowledge that the cohort was not restricted to patients at diagnostic onset, a design choice that ensured adequate sample size for our prospective 2-year follow-up. MFIS and BDI-II were collected at 6 months, introducing temporal overlap with outcome ascertainment, and CSF biomarkers were available only at diagnosis. Serum GFAP and quantitative MRI markers of compartmentalized activity (slowly expanding lesion volume, paramagnetic rim lesion count, and volumetric atrophy) were not available. Finally, the amount of missing data was significant, especially for the fluid biomarkers at baseline and PROs, and even though this was addressed using single median imputation, this may still have introduced bias. In contrast, predictors were collected systematically over 2 years through standardized clinical, biomarker, and patient-reported assessments at uniform time points, providing internally consistent longitudinal data within a well-characterized cohort. The outcomes were reassessed at a single 5-year time point, ensuring that the disability events observed during the 2-year follow-up and analyzed reflect durable progression rather than transient fluctuations or measurement noise.

Our findings contribute to a growing body of work showing that multimodal CSF and blood biomarker panels can prospectively stratify PIRA risk in early MS and provide a clinically accessible composite integrating a routine CSF neuroinflammatory marker with a patient-reported fatigue scale. Candidate predictors from univariate screening (CSF CHI3L1, MFIS, ΔSDMT, ΔT25FW, OCB positivity, and any new T2 lesion) represent a widely available and feasible starting set that requires validation in an adequately powered external cohort to assess discrimination, calibration, and generalizability before clinical implementation. Such validation will also benefit from harmonized PIRA definitions (38) to ensure comparability across studies.

## Data Availability

The raw data supporting the conclusions of this article will be made available by the authors, without undue reservation.
