# Supplementary material for: A 2-year multimodal prospective cohort study to predict sustained progression independent of relapse activity in multiple sclerosis
Source: Front Neurol. 2026 Oct 13;17:1902455. doi: 10.3389/fneur.2026.1902455 (PMC13710352; doi:10.3389/fneur.2026.1902455)
Supplement: Supplementary file 1 [file Supplementary_file_1.DOCX]

**Table S1 – Characteristics of excluded vs included patients in the full analysis**

| **Baseline variable** | **Full cohort (n = 129)** | **Included (n=81)** | **Excluded (n=48)** | **p-value** |
| --- | --- | --- | --- | --- |
| **Sex** |  |  |  | 0.4 |
| Female | 90 (70%) | 59 (73%) | 31 (66%) |  |
| Male | 38 (30%) | 22 (27%) | 16 (34%) |  |
| Missing | 1 | 0 | 1 |  |
| **Age at baseline (years)** | 32 [27, 37] | 32 [27, 37] | 33 [27, 38] | 0.5 |
| Missing | 3 | 0 | 3 |  |
| **Disease duration (months)** | 55 [27, 97] | 59 [35, 97] | 43 [14, 96] | 0.087 |
| Missing | 3 | 0 | 3 |  |
| **EDSS at baseline** | 1.50 [1.00, 2.50] | 2.00 [1.00, 2.50] | 1.50 [1.00, 2.00] | 0.028 |
| **DMT type at entry** |  |  |  | 0.10 |
| No DMT | 3 (2.4%) | 1 (1.2%) | 2 (4.3%) |  |
| High efficacy | 83 (65%) | 58 (72%) | 25 (54%) |  |
| Low/moderate efficacy | 41 (32%) | 22 (27%) | 19 (41%) |  |
| Missing | 2 | 0 | 2 |  |
| **Myelitis at onset** | 81 (65%) | 54 (67%) | 27 (63%) | 0.7 |
| Missing | 5 | 0 | 5 |  |
| **CSF CHI3L1 (pg/mL)** | 125 [84, 180] | 136 [88, 199] | 88 [63, 128] | 0.036 |
| Missing | 56 | 25 | 31 |  |
| **OCB status** |  |  |  | 0.8 |
| Negative | 15 (17%) | 11 (18%) | 4 (14%) |  |
| Positive | 74 (83%) | 49 (82%) | 25 (86%) |  |
| Missing | 40 | 21 | 19 |  |
| **Serum NfL at baseline (pg/mL)** | 6.9 [5.2, 9.1] | 6.5 [4.9, 8.3] | 7.8 [5.8, 10.4] | 0.027 |
| Missing | 20 | 12 | 8 |  |
| **MFIS total score (V2)** | 22 [7, 38] | 17 [6, 38] | 32 [10, 46] | 0.2 |
| Missing | 43 | 21 | 22 |  |
| **BDI-II score (V2)** | 8 [3, 21] | 8 [3, 21] | 8 [5, 18] | 0.7 |
| Missing | 70 | 31 | 39 |  |
| **Number of serum NfL samples** | 6 [4, 7] | 7 [6, 8] | 4 [3, 5] | <0.001 |

| **Table S2. Missing data (N = 81)** | | | |
| --- | --- | --- | --- |
| **Variable** | **N available** | **N missing** | **Missing (%)** |
| **Baseline clinical and biomarker variables** |  |  |  |
| Age at baseline (years) | 81 | 0 | 0.0% |
| Disease duration (months) | 81 | 0 | 0.0% |
| EDSS at baseline | 81 | 0 | 0.0% |
| Myelitis (yes/no) | 81 | 0 | 0.0% |
| CSF NfL | 52 | **29** | **35.8%** |
| CSF CHI3L1 | 56 | **25** | **30.9%** |
| IgG OCB | 60 | **21** | **25.9%** |
| mRio score | 80 | **1** | **1.2%** |
| Mean serum NfL (pg/mL) | 81 | 0 | 0.0% |
| Max serum NfL (pg/mL) | 81 | 0 | 0.0% |
| **Longitudinal changes (Δ last − baseline)** |  |  |  |
| ΔSDMT | 77 | **4** | **4.9%** |
| Δ9HPT dominant | 77 | **4** | **4.9%** |
| Δ9HPT non-dominant | 76 | **5** | **6.2%** |
| ΔT25FW | 74 | **7** | **8.6%** |
| ΔsNfL | 77 | **4** | **4.9%** |
| **MRI activity during follow-up** |  |  |  |
| New T2 lesions (years 1–2, sum) | 79 | **2** | **2.5%** |
| Any new T2 during follow-up (yes/no) | 79 | **2** | **2.5%** |
| **Patient-reported outcomes (V2, 6 months)** |  |  |  |
| MFIS total score | 60 | **21** | **25.9%** |
| Beck Depression score | 50 | **31** | **38.3%** |

*Missing values were handled by median imputation in all multivariable models. CSF biomarkers were available only in patients diagnosed in our center (diagnostic lumbar puncture). For longitudinal change variables, baseline was defined as V1 (or V2 for sNfL when V1 was unavailable) and the last measurement as V7 (or V8 for sNfL when available). Patient-reported outcomes (MFIS, BDI-II) were collected at V2 (month 6) according to protocol.*

**Table S3 – Sensitivity internal analysis of the reported model**

| **Model** | **EPV** | **Apparent AUC (95% CI)** | **Cross-validated AUC** | **Optimism** | **Calibration slope** |
| --- | --- | --- | --- | --- | --- |
| **Model reported in Table 3** | 2.6 | 0.86 (0.76-0.96) | 0.75 | 0.11 | 0.76 |
| **Pre-specified MFIS + CSF CHI3L1** | 5.3 | 0.81 (0.68-0.94) | 0.76 | 0.05 | 0.82 |
| **Screening with OCB excluded** | 3.5 | 0.83 (0.72-0.95) | 0.74 | 0.09 | 0.74 |
| **Imputation and scaling within folds** | 3.5 | 0.83 (0.72-0.95) | 0.75 | 0.09 | 0.75 |

*EPV, events per variable. Apparent AUC is the in-sample estimate with its DeLong 95% confidence interval. The cross-validated AUC is the median across 20 repeats of stratified 5-fold nested cross-validation, with variable selection performed entirely within each training fold. Optimism is the difference between the apparent and cross-validated estimates.*

**Figure S1. Schematic representation of the desired assessments and timepoints during the study time.**

*Figure S1. Chronological and conceptual representation of the full follow-up period of the study as per protocol*. 9HPT: 9-Hole Peg Test; CHI3L1: Chitinase-3-like-1 protein 1; EDSS: Expanded Disability Status Scale; FLAIR: Fluid-Attenuated Inversion Recovery; GRE: Gradient Echo; NFL: Neurofilament Light Chain; OCB: Oligoclonal Bands; RRMS: Relapsing-Remitting Multiple Sclerosis; SDMT: Symbol Digit Modalities Test; SPMS: Secondary Progressive Multiple Sclerosis; SWI: Susceptibility Weighted Imaging; T25FW: Timed 25-Foot Walk.*

** Note: not all patients underwent through all assessments and with the exact time period represented in this figure. Due to causes outside of the study team control (e.g., COVID-19 pandemic) some of the assessment could have been missed for some of the patients.*
